# *Aonchotheca annulosa* and *Aonchotheca murissylvatici*, which is which? A reappraisal of the gastrointestinal *Aonchotheca* (Nematoda: Capillariidae) species common in wood mice and bank voles

**DOI:** 10.1017/S0031182024001471

**Published:** 2024-12

**Authors:** Jerzy M. Behnke, Joseph A. Jackson

**Affiliations:** 1 School of Life Sciences, University of Nottingham, University Park, Nottingham, UK; 2 School of Science, Engineering and Environment, University of Salford, Manchester, UK

**Keywords:** *Aonchotheca annulosa*, *Aonchotheca halli*, *Aonchotheca murissylvatici*, *Apodemus* spp, *Myodes glareolus*, small intestine, spicule, stomach

## Abstract

Wood mice (*Apodemus sylvaticus*) and bank voles (*Myodes glareolus*) are often employed as natural study models in infectious disease ecology. Yet the identities of some elements of their parasite fauna have been subject to long-standing confusion. One instance of this relates to 2 nominal species of the capillariid nematode genus *Aonchotheca*: *Aonchotheca annulosa* (Dujardin, 1845) and *A. murissylvatici* (Diesing, 1851). Through literature review, analysis of recorded host- and site-specificity and tracing of taxonomic precedence, it is possible to confirm that *A. annulosa* is a valid species with a spicule c. 1000 microns long, a small intestinal site of infection and a wide host range centred in murine rodents (with *A. sylvaticus* the most common host). On the other hand, tracing the provenance of *A. murissylavtici* through to the works of the early naturalists reveals it is best assigned as a *nomen nudum* (lacking sufficient establishing description) or a junior synonym of *A. annulosa* and does not have precedence for the other *Aonchotheca* morphotype commonly found in Eurasian rodents. The first description consonant with this other morphotype, which has a short spicule (200–250 microns in length) and occurs primarily in the stomach of bank voles and other cricetids, was as *Capillaria halli* by Kalantarian in 1924. We thus recommend the suppression of *A. murissyvatici* in favour of *Aonchotheca halli* (Kalantarian, 1924) for this gastric-specialist short-spicule morphotype, particularly as the use of the *A. murissylvatici* name and its variants has previously been associated with substantial inconsistency and misidentification with *A. annulosa*.

## Graphical abstract

**Figure:**
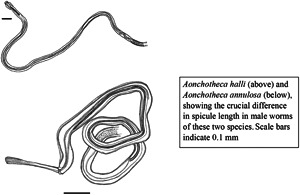

## Introduction

Wood mice (*Apodemus sylvaticus*) and bank voles (*Myodes glareolus*) have often been employed by parasitologists and infectious disease ecologists as natural study models. Despite this relatively high level of attention, however, the identities of some elements of their parasite fauna have been subject to long-standing confusion. One instance of this relates to 2 nominal *Aonchotheca* species that have frequently been reported in the gastrointestinal lumen of wood mice and bank voles, and in other Eurasian rodents. These are *Aonchotheca annulosa* (Dujardin, 1845) and *A. murissylvatici* (Diesing, 1851). Unfortunately, although 2 genuine species do seem to be involved, these appear to have been confused for one another in a substantial proportion of the literature, especially that relating to epidemiology and helminth community structure in wild European rodents.

*Aonchotheca* spp. are nematodes belonging to the trichinelloid family Capillariidae Railliet, 1915 which contains several species of medical or veterinary significance. Capillariids parasitize all 5 classes of vertebrates and comprise over several hundred described species (Moravec, 2000), each of which specializes in exploiting a particular host body organ (e.g. intestinal tract, liver, bladder, etc.). Capillariid life cycles can be complex, involving up to 2 different intermediate, transport or paratenic hosts, but few have been documented in detail, and the range of hosts of some species is poorly known (Moravec *et al*., 1987; Anderson, 2000). The taxonomy of this group is also complex, with many changes over the years of the scientific nomenclature of individual species, as well as revision of the structure of the taxon (Moravec, 1982, 2000). Moreover, the recent application of molecular tools has revealed cases of incongruence with phylogenetic studies based on classic morphology (Borba *et al*., 2019; Deng *et al*., 2022).

Capillariids frequently adopt histozoic infection habits, burrowing in the solid tissue of various organs (Anderson, 2000), and this may lead to an increased propensity to cause disease. For example, the species considered here often burrow partially within the gastrointestinal mucosa (Roman, 1951). Sometimes they are associated with tumour-like formations in the stomach (Roman, 1951) or may build up to high population sizes, forming tangled aggregations that could physically interfere with digestion. Furthermore, a number of the better-studied capillariids lack narrow specificity to the definitive host and may cause transboundary or zoonotic outbreaks. Examples of this are *Paracapillaria philippinensis*, the agent of intestinal capillariasis (Lu *et al*., 2006), *Calodium hepaticum*, the agent of hepatic capillariasis (Fuehrer *et al*., 2011), and *Eucoleus aerophilus*, a lungworm primarily infecting canids that can also infect humans (Lalosević *et al*., 2008). Even amongst the species considered here, *A. annulosa* is known to infect primates (Capuchin Monkeys and Baboons) in captivity and thus might have some zoonotic potential (Moravec and Baruš, 1991). Given these disease-causing and host-switching proclivities, and the past confusion that has affected the identification of the common gastrointestinal *Aonchotheca* nematodes occurring in rodents in Eurasia, our aim here is to clarify the systematics and nomenclature of these species.

Below we begin by reviewing the historic literature on *Aonchotheca*-like forms in the gastrointestinal lumen of wood mice, bank voles and other Eurasian rodents and then we identify the problems that have given rise to confusion. Finally, we propose a solution that is biologically representative and that also conforms to the rules of Zoological nomenclature, and we recommend how these species should be identified and referred to in future work.

## *Aonchotheca annulosa* is a valid species

The earliest record of capillariid nematodes from wild rodents is by Dujardin (1845), who described a species from the intestine of rats in Northern France, the males of which possessed a relatively long spicule (0.95 mm). Dujardin named these nematodes *Calodium annulosum*. Travassos (1915) moved the species to the genus *Capillaria*, as *Capillaria annulosa*, and then López-Neyra (1947) to *Aonchotheca*. Hence this species is now usually known as *A. annulosa*. The relatively long spicule of *A. annulosa* has been reaffirmed on multiple occasions (e.g. Mészáros, 1977 [1.00–1.02 mm]; Mészáros, 1978 [1.00 mm]; Bain and Wertheim, 1981 [1.14 mm]; Moravec and Baruš, 1991 [1.11–1.27 mm]; Umur *et al*., 2012 [0.86–1.08 mm]) in consonant material. Thus, the modern concept of *A. annulosa* is clearly linked to Dujardin's original record by the presence of a distinctive long spicule and by the typical site of infection in the small intestine of Eurasian rodents. *Aonchotheca annulosa* may therefore be considered an uncomplicated and valid taxon.

## *Aonchotheca halli* has precedence on a short-spicule morphotype mostly occurring in Eurasian cricetids

In the same monograph of 1845, Dujardin also described female worms from the intestine (Diesing, 1851) of *Mus sylvaticus* (= *A. sylvaticus*) which he referred to as ‘Trichosome du mulot (*Mus sylvaticus*)’. Six years later Diesing (1851) created the name *Trichosomum muris sylvatici* Dujardin, based on some part of the specimens collected by Dujardin from wood mice. Diesing specified Dujardin as the authority for this taxon, but as Dujardin did not formally name his specimens, Diesing has been taken as the authority by subsequent authors. Diesing (1851) provided only very sparse and ambiguous information to add to the minimal description provided by Dujardin, and together these works lack any diagnostic morphological information useful in distinguishing between the *Aonchotheca* morphotypes found in present-day Eurasian rodents. In particular, no information was provided by either author on male worms, which were lacking in Dujardin's original collection. In all likelihood, Diesing's taxon was the same species, *A. annulosa*, as the specimens Dujardin had found in the intestine of rats, given the preference of *A. annulosa* for the small intestine of murine hosts (see also below). Thus, *T. muris sylvatici* of Diesing might be regarded as a junior synonym of *A. annulosa*. Alternatively, it could be argued that the information surrounding *T. muris sylvatici* is so sparse and inconclusive that it should instead be considered a *nomen nudum* (i.e. effectively lacking an establishing description [International Commission on Zoological Nomenclature, 1999]).

Despite its poor support in evidence, *T. muris sylvatici* Diesing (1851) was moved to the genus *Capillaria Z*eder, 1800 by Travassos (1915; see also Moravec, 1982). Thereafter it became stabilized within *Capillaria* or *Aonchotheca* (which is the generic classification we accept here) by later authors who arbitrarily linked it to small-spicule morphotypes that were not described for the first time until the early 20th century. At times 3 different versions of the specific name have been employed: *muris sylvatici*, *muris-sylvatici* or *murissylvatici*. In fact, the earliest record of a rodent-infecting *Aonchotheca*-like capillariid clearly different to *A. annulosa* was by Kalantarian (1924) who described material with a short spicule (0.1928 mm; approximately one-quarter of the length found in *A. annulosa*), that she recovered from the migratory or grey dwarf hamster (*Nothocricetulus migratorius* (= *Cricetulus migratorius*) [Pallas, 1773]) in Armenia. This was named *Capillaria halli* by Kalantarian (1924) but was later synonymized with *C. murissylvatici*, by Teixeira De Freitas and Lent (1936). These latter authors gave the spicule length for *C. murissylvatici* as exactly the same as given earlier by Kalantarian (1924) for *C. halli*, although they did not attribute this measurement to her work. Teixeira De Freitas and Lent (1936) included bank voles and wood mice, alongside the migratory hamster, as hosts of *C*. *murissylvatici*, likely on the basis of records by Baylis (1928) and Elton *et al*. (1931) mentioned further below.

Following the work of Teixeira De Freitas and Lent (1936), reports of *C. halli*-like short-spicule morphotypes tended to be attributed to *C. murissylvatici*. Roman (1939) reported worms occurring in large numbers within tumour-like developments of the gastric mucosa of French voles (primarily bank voles). He provided comprehensive measurements of gastric specimens from bank voles that confirmed the relatively short spicule of the male worms (0.187–0.247 mm and mean of 0.219 mm). In this publication and in his review of 1951 (Roman, 1951) Roman further established one concept of *C. murissylvatici* as a parasite of the gastric mucosa of microtine voles. This concept was followed by several later authors, who placed the species either in *Capillaria* or *Aonchotheca*. This included Justine and de Roguin (1990) who again confirmed the short spicule length (0.200–0.215 mm) in stomach-dwelling forms.

## *Aonchotheca annulosa* in the small intestine has likely often been misidentified

*Aonchotheca* nematodes are long, thin, filamentous worms that penetrate the intestinal mucosa. Because of their fragility, their length and their location in the mucosa, they are easily fragmented upon recovery from the host. Moreover, they are not easy to identify precisely by those who are not closely familiar with the taxon. It is perhaps not surprising, therefore, that additional confusion has arisen in the literature about whether the worms observed in rodent intestines were *A. murissylvatici* or *A. annulosa*. This confusion has been further exacerbated by reports in journals that are not easily accessed even today (especially those from East European university and society journals), and hence relevant studies have not always been available to those describing and/or reporting on these species.

The stage for confusion between the stomach and small intestine-specialist *Aonchotheca* species may have been partly set by Elton *et al*. (1931). Following Baylis (1928) these authors reported what they called ‘*Capillaria*? *muris-sylvatici*’ from the small intestines of both wood mice and bank voles. Given the site of infection, this is in fact likely to have been *A. annulosa*. Furthermore, Elton *et al*. (1931) reported ‘*Capillaria* or *Hepaticola sp.indet.*’ from the stomachs of wood mice, but, given the site of infection, this is likely to have been the *A. halli* morphotype associated with *A. murissylvatici* by many authors (see above), or a *Eucoleus* species. Several authors working on the epidemiology of helminths in wild rodents from the British Isles (Lewis, 1968*a*; Canning *et al*., 1973; Langley and Fairley, 1982; O'Sullivan *et al*., 1984; Montgomery and Montgomery, 1988, 1990; Abu-Madi *et al*., 2000; Loxton *et al*., 2016; Stuart *et al*., 2020), as well as those working in continental Europe (Sołtys, 1949; Erhardová and Ryšavý, 1955; Tenora and Baruš, 1955; Tenora and Zavadil, 1967; Bjelić-Čabrilo *et al*., 2009, 2011; Movsesyan *et al*., 2018) followed this lead, reporting *A. murissylvatici* from the small intestines of rodents (see Table 1). Thus, although *A. annulosa* has been recorded only rarely in the British Isles (James, 1954; Wakelin, 1968; Jackson *et al*., 2009; Behnke unpublished observations) this likely reflects underreporting due to misidentification with *A. murissylvatici*. On the European mainland, despite some likely misidentification (discussed by Moravec [2000]), *A. annulosa* is nonetheless recognized as a frequent parasite of the small intestine from studies of bank voles (Tenora and Zejda, 1974; Mészáros, 1978; Milazzo *et al*., 2003*a*; Grzybek *et al*., 2015), and of other rodent species (Moravec, 2000).

**Table 1.** Chronological list of papers reporting *Aonchotheca murissylvatici*

Author (date)	Host	Location	*Aonchothecas* sp.^a^	Intestinal site	Prevalence and mean abundance or range
Diesing (1851)	*A. sylvaticus*	not stated	*Trichosomum muris sylvatici*	Intestine	Not stated
Travassos (1915) (review)	*A. sylvaticus*	not stated	*Capillaria muris-sylvatici*	Not stated	Not stated
Baylis (1928)	*A. sylvaticus*	Oxfordshire	*Capillaria*? *muris-sylvatici*	Not stated	Not stated
	*M. glareolus*		*Capillaria*? *muris-sylvatici*	Not stated	Not stated
Elton *et al*. (1931)	*A. sylvaticus*	Oxfordshire	*Capillaria*? *muris-sylvatici*	Small intestine	4% and low
	*A. sylvaticus*		*Capillaria*? *Hepaticola sp.indet*	Stomach	0.1% and low
	*M. glareolus*		*Capillaria*? *muris-sylvatici*	Small intestine	Rare and low
Teixeira De Freitas and Lent (1936) (review)	*A. sylvaticus*	Europe	*Capillaria muris-sylvatici*	Intestine	Not stated
	*N. migratorius*				
	*M. glareolus*				
Baylis (1939)	*A. sylvaticus*	Westmorland	*Capillaria*? *muris-sylvatici*	Not stated	Not stated
Roman (1939)	*M. glareolus*	France	*Capillaria muris-sylvatici*	**Stomach**	Not stated
Read (1949)	*M. p. pennsylvanicus*	USA	*Capillaria muris-sylvatici*	Small intestine	Not stated
Sołtys (1949)	*A. flavicollis*	Poland	*Capillaria muris-sylvatici*	Small intestine	Not stated
	*M. glareolus*				
Roman (1951)	*M. glareolus*	France	*Capillaria muris-sylvatici*	**Stomach**	Not stated
Thomas (1953)	*A. sylvaticus*	Scotland	*Capillaria muris-sylvatici*	**Stomach**	37.8% and low
	*M. glareolus*				64.3% and high
James (1954)	*A. sylvaticus*	W. Wales	*Capillaria muris-sylvatici*	Not stated	Not stated
	*M. glareolus*				
Erhardová and Ryšavý (1955)	*Apodemus* spp.	Czechia	*Capillaria muris-sylvatici*	Small intestine	Not stated and 1–22
	*Microtus arvalis*				
	*Myodes glareolus*				
Tenora and Baruś (1955)	*A. flavicollis*	Czechia	*Capillaria muris-sylvatici*	Small intestine	2.1% and 0–36
Sharpe (1964)	*A.sylvaticus*	Bristol	*Capillaria muris-sylvatici*	Not stated	Low and rare
Tenora and Zavadil (1967) (review)	*Apodemus* spp.		*Capillaria murissylvatici*	Small intestine	Not stated
	*Microtus arvalis*.				
	*Myodes glareolus*				
	*Rattus* spp.				
Lewis (1968*a*)	*A. sylvaticus*	W. Wales	*Capillaria muris sylvatici*	Small and large intestine	1.4% and 1–5
Lewis (1968*b*)	*M. glareolus*		*Capillaria muris sylvatici*	**Stomach** and small intestine	14.9% and 4–187
Kisielewska (1970*a*, 1970*b*)	*M. glareolus*	E. Poland	*Capillaria muris sylvatici*	**Stomach**	17.1–32.2% and 1.0–12.9
Kisielewska (1983)	*M. glareolus*		*Capillaria muris sylvatici*	**Stomach**	17.1–32.2% and 1.0–12.9
Lewis and Twigg (1972)	*M. glareolus*	Surrey	*Capillaria muris sylvatici*	**Stomach** and small intestine	69.3% and 1–96^b^
Canning *et al*. (1973)	*M. glareolus*	S. Devon	*Capillaria muris sylvatici*	Intestine	Common
	*A. sylvaticus*				
Tenora and Zejda (1974)	*M. glareolus*	Czechoslovakia	*Capillaria muris-sylvatici*	Small intestine	Not stated
Murúa (1978)	*M. glareolus*	Bristol	*Capillaria muris-sylvatici*	**Stomach**	92% and 189.1
	*A. sylvaticus*		*Capillaria* spp.	Small intestine	5% and 0.25
Langley and Fairley (1982)	*A. sylvaticus*	W Ireland	*Capillaria muris sylvatici*	Small intestine	10% and 0–113
O'Sullivan *et al*. (1984)	*M. glareolus*	SW Ireland	*Capillaria muris-sylvatici*	Small intestine	32% and 0–96
	*A. sylvaticus*				12% and 0–7
Montgomery and Montgomery (1988)	*A. sylvaticus*	N. Ireland	*Capillaria murissylvatici*	Small intestine^c^	0–75% and 5.8
Montgomery and Montgomery (1990)	*A. sylvaticus*		*Capillaria murissylvatici*	Small intestine^c^	0–75% and 5.8
Feliu *et al*. (1997)	*A. sylvaticus*	Spain	*Aonchotheca muris-sylvatici*	Not stated	Not stated
	*M. glareolus*				
	*R. norvegicus*				
Tenora *et al*. (1977)	*A. sylvaticus*	Norway	*Capillaria muris-sylvatici*	Small intestine	2.0%
Behnke *et al*. (1999)	*A. sylvaticus*	Surrey	*Capillaria murissylvatici*	Not stated	8.2% and 0–18
Abu-Madi *et al*. (2000)	*A. sylvaticus*	Sussex	*Capillaria murissylvatici*	Small intestine^c^	2.5% and 0–8
Moravec (2000) (review)	*Apodemus* spp.		*Aonchotheca murissylvatici*	Small intestine	Not stated
	*Microtus* spp.				
				Rarely in stomach	
	*Myodes* spp.				
Milazzo *et al*. (2005)	*A. sylvaticus*	Italy	*Aonchotheca muris-sylvatici*	Intestine (not stomach)	1.9% and 1–24
Klimpel *et al*. (2007)	*M. glareolus*	Germany	*Aonchotheca murissylvatici*	Digestive tract	51.7% and 2–204
Bjelić-Čabrilo *et al*. (2009)	*M. glareolus*	Serbia	*Capillaria murissylvatici*	Small intestine	25% and 1–13
Ribas *et al*. (2009)	*M. glareolus*	Spain	*Aonchotheca muris-sylvatici*	Not stated	1.1% and 1–2
Bjelić-Čabrilo *et al*. (2011)	*M. glareolus*	Serbia	*Capillaria murissylvatici*	Small intestine	16.4% and 1–271
Knowles *et al*. (2013)	*A. sylvaticus*	Cheshire	*Aonchotheca murissylvatici*	Not stated	1.7% and not given
Bjelić-Čabrilo *et al*. (2013)	*A. sylvaticus*	Serbia	*Capillaria murissylvatici*	Intestine	2.27% and 0.05
Loxton *et al*. (2016)	*M. glareolus*	Ireland	*Aonchotheca murissylvatici*	Small intestine^c^	28.2% and 33.1
	*A. sylvaticus*				15.8% and 10.2
Loxton *et al*. (2017)	*A. sylvaticus*	Ireland	*Aonchotheca murissylvatici*	Small intestine	9.77% and 0.2/1.6
Movsesyan *et al*. (2018) (Review)	*A. uralensis*	Armenia	*Aonchotheca murissylvatici*	Small intestine	Not stated
	*N. migratorius*				
	*M. musculus*				
	*M. arvalis*				
Stuart *et al*. (2020)	*M. glareolus*	Ireland	*Aonchotheca murissylvatici*	Large intestine	19.3% and 2.9
	*A. sylvaticus*				13.7% and 1.9
Behnke *et al*. (2021)	*A. sylvaticus*	Yorkshire	*Aonchotheca murissylvatici*	Not stated	6.4% and 0.34
Miljević *et al*. (2022)	*M. glareolus*	Serbia	*Aonchotheca murissylvatici*	**Stomach**^c^	9.5% and 3.3
Lewis *et al*. (2023)	*A. sylvaticus*	S. England	*Aonchotheca murissylvatici*	Not stated	0.2% and <0.01

For each publication, we give the name of the parasite as stated in that paper. Note, that this is not intended to be a comprehensive list of all papers, but the majority of studies undertaken in the British Isles are covered, and representative/key studies from abroad have also been included. We have not included all recent studies where no information was given on the criteria for species assignment nor on the gastrointestinal site from which the worms were recovered. Initially, the species was recorded as *Trichosomum muris sylvatici* by Diesing (1851). It was then moved to the genus *Capillaria Z*eder, 1800 by Travassos (1915), and subsequently to a new genus, *Aonchotheca*, by López-Neyra in 1947, although as is evident above, for over 60 further years many authors continued to refer to *Capillaria murissylvatici*. The hosts reported above are: *Apodemus sylvaticus*, *Apodemus uralensis*, *Rattus norvegicus*, *Nothocricetulus migratorius* (syn. *Cricetulus accedula*), *Microtus arvalis*, *Microtus pennsylvanicus* and *Myodes glareolus*. *Clethrionomys* is taken to be a junior synonym of *Myodes* and records previously named *Cricetulus migratorius* are assigned to *Nothocricetulus migratorius*.

a The original version of the name as in publication.

b Not stated whether the high frequency and intensity were for worms in the stomach or small intestine. Sympatric wood mice were not infected.

c In these studies the intestinal site is not given in the paper but has been confirmed by correspondence with the authors.

In contrast to authors who understood *A. murissylvatici* to be a small intestinal parasite, others, following Texeira De Freitas and Lent (1936) and Roman (1951), were quite definite that *A. murissylvatici* is a parasite of the stomach. Among the earliest was Thomas (1953) who found heavy infections in the stomachs of bank voles in Scotland, and then the work of Kisielewska (1970*a*, 1970*b*, 1983) in Poland emphasized that *A. murissylvatici* is a bank vole stomach specialist. Others include Murúa (1978), Justine and de Roguin (1990) and more recently, Miljević *et al*. (2022).

## Other relevant *Aonchotheca* species in Eurasia

*Aonchotheca annulosa* and *A. halli* are the only *Aonchotheca* species to be frequently reported in Eurasian rodents (with nominal records of *A. murissylvatici* most likely to be one or the other), if *A. wioletti* (Rukhlyadeva, 1950) is accepted as a junior synonym of *A. halli* (following Justine and de Roguin, 1990). However, an assemblage of *Aonchotheca*-like species is known in glirids (dormice) (Justine *et al*., 1987; Veciana *et al*., 2016) whose members could potentially be confused with *A. halli* and should be kept in mind given the low specificity of many capillariids. Of these forms in glirids, both *A. myoxinitelae* (Diesing, 1851) and *A. legerae* (Justine *et al*., 1987) appear to have a longer spicule than *A. halli* (>0.26 mm). Furthermore, both have more prominent structures around the vulval opening and shorter ejaculatory ducts (Pisanu and Bain, 1999), amongst other potential differentiating features (Justine *et al*., 1987; Pisanu and Bain, 1999). *Tenoranema alcoveri* Mas-Coma and Esteban, 1985, also from glirids, which is close to *Aonchotheca* and whose generic characters require further clarification, has a spicule that overlaps that of *A. halli*, but is distinguished by the presence of complex digitiform rays supporting the bursa (Mas-Coma and Esteban, 1985; Justine *et al*., 1987). Apart from these species in glirids, 2 additional rodent-infecting species occur in Eurasia. This includes the relatively poorly known *A. armeniaca* (Kirschenblat, 1939), which has a long spicule (1.10 mm) and infects the small intestine of *Citellus* spp. in Armenia (Veciana *et al*., 2016), and the more recently described *Aonchotheca yannickchavali*, Veciana *et al*., 2016 which is distinguished by a very large, tube-like projection associated with the vulva and infects the intestine of *Bandicota* species in Thailand (Veciana *et al*., 2016). Moreover, it should be borne in mind that several poorly known species with some similarity to *A. halli* have been recorded in a variety of non-rodent mammalian hosts in Eurasia. This includes *A. musimon* Pisanu and Bain, 1999 and *A. bilobata* (Bhalerao, 1933) in ungulates and *A. speciosa* (Beneden, 1873) in bats. The existence of this additional sympatric diversity emphasizes the importance of future molecular studies to further elucidate the relationships of *A. halli* to similar forms in non-rodent hosts.

## Recommendations

In order to survive in the hostile environment of the mammalian intestine, helminths evolve to become specialists in parasitizing specific regions of the intestine where they can best resist acidity, host enzymes and other defences against invasion by microorganisms (Schad, 1963; Crompton, 1973; Sukhdeo and Sukhdeo, 1994; Sukhdeo and Bansemir, 1996). In our view it is unlikely, therefore, that a species that has become a specialist for survival in the duodenum, will be able to cope equally well in the stomach.

As justified in the sections above, the most parsimonious explanation of the confusion in the literature is that the commonly-occurring gastrointestinal *Aonchotheca* fauna of European rodents is made up of 2 site-specialist species. One is a gastric mucosal specialist, living intertwined in the glandular, pyloric region of its host's stomach and while perhaps occasionally worms in heavy infections may spill over into the duodenum, this nematode is not usually a resident of the small intestine. The other is an intestinal specialist species, *A. annulosa*, also usually with a mucosal burrowing habit. We propose that most of the previous reports of ‘*A. murissylvatici*’ from the small intestine were in fact *A. annulosa*. We further propose that the stomach-dwelling species should be referred to by the name *Aonchotheca halli* (Kalantarian, 1924). As explained above, this has precedence on the stomach-dwelling short-spicule, cricetid-specialist morphotype, replacing ‘*A. murissylvatici*’ which has very dubious support in evidence. Although the type specimens of *A. halli* and its original description are not available (despite our efforts to locate these), Texeira De Freitas and Lent's redrawings of Kalantarian's specimens are sufficient to link these to the small-spicule gastrointestinal *Aonchotheca* morphotype occurring in Eurasian cricetids. This shift in nomenclature is in line with rules on precedence in the International Zoological Code of Nomenclature (International Commission on Zoological Nomenclature, 1999). Moreover, rather than disturbing stability and precedent, this in fact draws a line under more than 100 years of incorrect, inconsistent and biologically confusing use of the name *A. murissylvatici* and its variants, within which a large proportion of nominal records are likely to have been misidentifications of *A. annulosa*. One additional advantage of suppressing A. *murissylvatici* will be that the instability associated with the exact form of the name (*muris sylvatici*/*muris-sylvatici*/*murissylvatici*) will also be curtailed.

## The host-specificity, geographical distribution, zoonotic potential and identification of *A. annulosa* and *A. halli*

**Figure 1.**
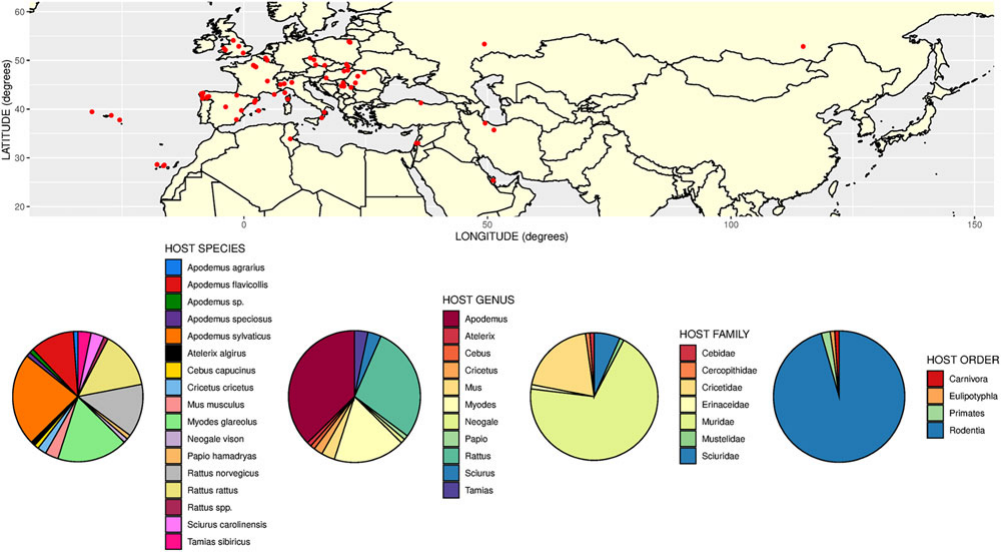
Geographical and host distribution of *Aonchotheca annulosa* (Dujardin, 1845). Top panel shows locations of nominal records of *A. annulosa* in Eurasia. Bottom panels show pie charts representing the proportional frequency of records of *A. annulosa* at different host taxonomic levels. Based on 91 records drawn from publications cited in the main text and also Balfour (1922), Lewis (1927), Bernard (1961), Mészáros and Murai (1979), Mas-Coma and Feliu (1981), Mészáros *et al*. (1983), Mészáros and Štollmann (1984), Jirouš (1985), Justine (1989), Mascato *et al*. (1993), Afonso-Roque (1995), Asakawa and Tenora (1996), Casanova *et al*. (1996), Milazzo *et al*. (2003*b*), Fuentes *et al*. (2004, 2010), Pisanu *et al*. (2007, 2009), Ondríková *et al*. (2010), Salvador *et al*. (2011), Kirillova (2011, 2012), Romeo *et al*. (2012, 2014), Debenedetti *et al*. (2014, 2015), López González (2014), Meshkekar *et al*. (2014), Čabrilo *et al*. (2016, 2018), Martínez-Rondán *et al*. (2017), Galán-Puchades *et al*. (2018), Mazhari *et al*. (2019), Islam *et al*. (2024). Points either represent records from specific localities or central points for a general area, depending on the precision given in the respective publications.

**Figure 2.**
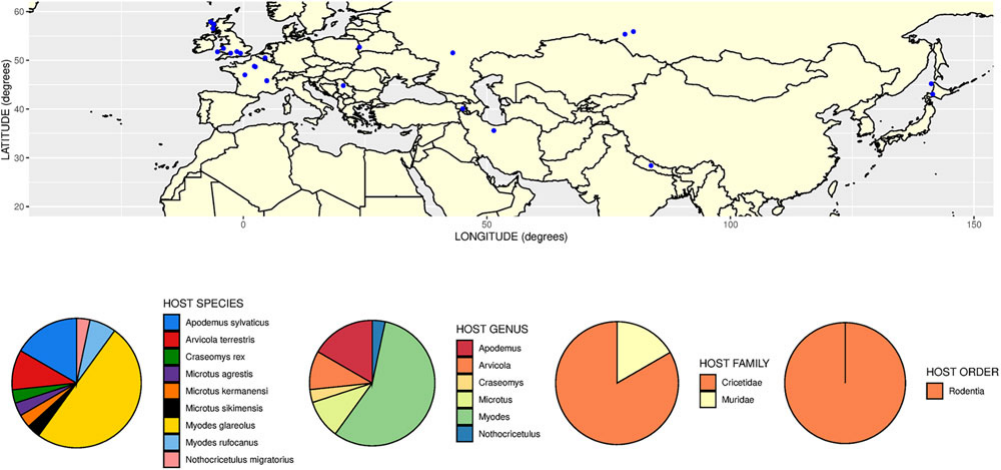
Geographical and host distribution of *Aonchotheca halli* (Kalantarian, 1924). Top panel shows locations of records of ‘*A. murissylvatici*’ where these were from the stomach, or where there was a definite morphological identification. Bottom panels show pie charts representing the proportional frequency of records of *A. halli* at different host taxonomic levels. Based on 30 records drawn from publications cited in the main text or Fig. 1 and also Asakawa *et al*. (1983, 1992, 1997).

Given clarification of the species concepts for these *Aonchotheca* morphotypes common in wood mice and bank voles, a clearer picture can be formed of their broader host and geographical distributions. To this end we carried out a literature search of host and geographical records. We accepted all records of *A. annulosa* from the intestine of hosts. For *A. halli*, we only accepted records of ‘*A. murissylvatici*’ from the stomach of hosts, or in the case of intestinal records, where a positive identification had been made based on morphological criteria. From summaries of the resulting data (Figs 1 and 2) it can be seen that *A. annulosa* (Fig. 1) has the wider host range of the 2 species. Whilst it is most often recorded in *Apodemus* and *Rattus* it can also infect a range of other murines but also cricetids, sciurids, mustelids and insectivores. Moreover, it has been recorded more than once in primates (baboons and monkeys) in zoological park settings (Moravec, 2000; Umur *et al*., 2012). As previously recognized, this wide host range could be indicative of transboundary and zoonotic potential. The distribution of this species extends across a very wide area of Eurasia, with records from Atlantic islands likely indicating a propensity to be spread anthropogenically *via* commensal murine hosts (e.g. rats). In contrast to *A. annulosa*, *A. halli* has narrower specificity infecting primarily bank voles, but also some other cricetids and, in fewer cases, murines (Fig. 2) . Although reliable records of this species are less numerous, it also seems to have a very wide distribution within Eurasia (Fig. 2).

**Figure 3.**
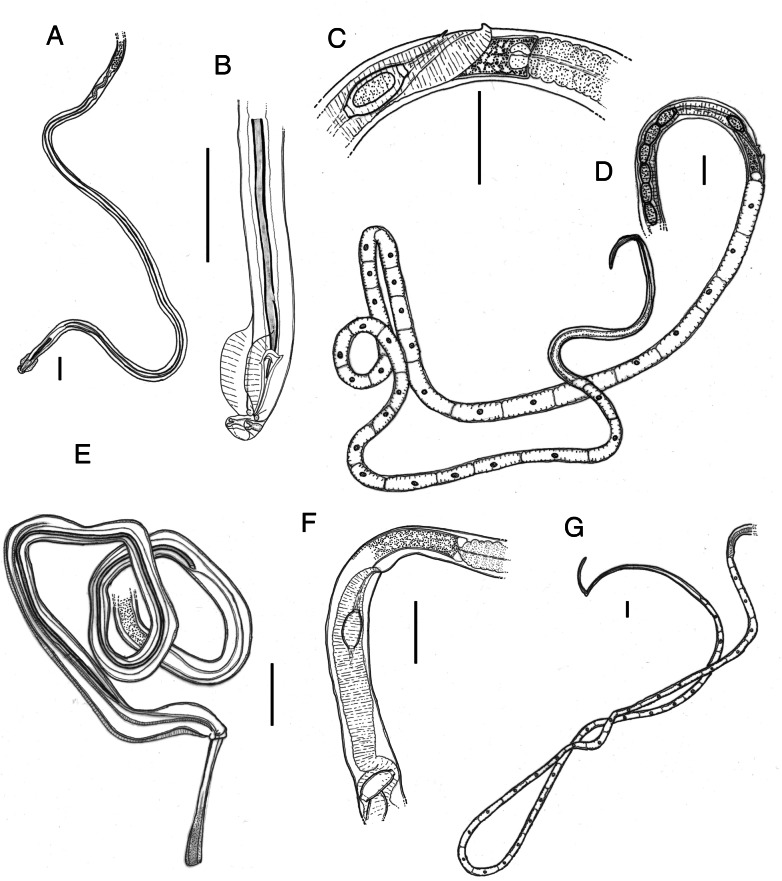
Diagnostic features in *Aonchotheca halli* (Kalantarian, 1924) (A–D) and *Aonchotheca annulosa* (Dujardin, 1845) (E–G). Scale bars indicate 0.1 mm. (A) Posterior region of male worm. (B) Posterior extremity of male worm with spicule in lateral view. (C) Terminal region of female reproductive tract. (D) Anterior region of female worm. (E) Posterior region of male worm, with partly evaginated spicule sheath, in sublateral view. (F) Terminal region of female reproductive tract. (G) Anterior region of female worm. A–D are based on specimens from the stomach of bank voles in Cornwall, UK; E–G are based on specimens from the intestine of wood mice (*Apodemus sylvaticus*) in Nottinghamshire, UK.

For the practical purposes of future identification, these 2 species differ in several morphological criteria (and accurate descriptions can be found in Moravec [2000] and Justine and de Roguin [1990]) but, if male worms are available, can be most easily distinguished by the difference in the length of spicules: *A. annulosa* has spicules that are about 4 times longer than those of *A. halli* (c. 0.20–0.25 *vs* c. 1.00 mm) (Table 2, Fig. 3). In females, *A. halli* has a small vulval appendage (Justine and de Roguin, 1990; see also Read, 1949) and *A. annulosa* lacks such an appendage but usually has an elevated anterior vulval lip (Moravec and Baruš, 1991) (Fig. 3). A key for the identification of the *Aonchotheca* species found in European rodents is provided in Table 2. For unambiguous differentiation of *A. annulosa* and *A. halli* that does not depend on skilled microscopic observation, the use of 18S rRNA gene DNA sequencing might be recommended. Our preliminary molecular results (manuscript in prep.) suggest this will easily distinguish between these 2 species. In a forthcoming study, we will be depositing reference sets of sequences for both species that will facilitate molecular identification.

**Table 2.** A dichotomous key for the identification of *Aonchotheca* species in European rodents

Parent node	Dichotomy	Child node
1. Capillariids from European rodents	Spicule sheath spiny (see Moravec, 1982)	**2**
	Spicule sheath non-spiny and well-developed bursa	**3**
2. *Eucoleus* spp. Predominantly infecting the stomach.	Egg length < 60 microns	***Eucoleus bacillatus***
	Egg length > 60 microns	***Eucoleus gastricus***
3. *Aonchotheca*-like species spp.	Bursa containing simple pedunculate papillae	**4**
	Bursa supported by a complex of digitiform rays	***Tenoranema alcoveri*** Only recorded in the small intestine of glirids. Genus-level characters require clarification
4. *Aonchotheca* spp.	Spicule short < 0.350	**5**
	Spicule long > 0.9	***Aonchotheca annulosa*** Infecting the small intestine
5. Short-spicule *Aoncotheca* spp. species	Spicule length < 0.260	***Aonchotheca halli*** Predominantly infecting the stomach
	Spicule length > 0.260	**6**
6. Short-spicule *Aonchotheca* spp. with spicule > 0.260	2 pairs of bursal papillae	***Aonchotheca legerae*** Only recorded in the small intestine of glirids
	1 pair of bursal papillae	***Aonchotheca myoxinitelae*** Amongst rodents, only recorded in the small intestine of glirids

*Note*: not included in the key, in wider Eurasia other records include the poorly known *A. armeniaca*, which has a spicule c. 1.1 mm in length and infects the small intestine of *Citellus* spp. and *A. yannickchavali* which infects the stomach of *Bandicota* spp. in Thailand and is distinguished by a very large tube-like projection associated with the vulva (see Veciana *et al*., 2016). Measurements are given in mm unless otherwise indicated.

## Data Availability

The authors confirm that the data supporting the findings of this study are available within the article.
